# Potassium Deficiency in Rice Aggravates *Sarocladium oryzae* Infection and Ultimately Leads to Alterations in Endophyte Communities and Suppression of Nutrient Uptake

**DOI:** 10.3389/fpls.2022.882359

**Published:** 2022-04-26

**Authors:** Jianglin Zhang, Zhifeng Lu, Rihuan Cong, Tao Ren, Jianwei Lu, Xiaokun Li

**Affiliations:** ^1^Key Laboratory of Arable Land Conservation (Middle and Lower Reaches of Yangtze River), College of Resources and Environment, Ministry of Agriculture and Rural Affairs, Microelement Research Center, Huazhong Agricultural University, Wuhan, China; ^2^Shuangshui Shuanglv Institute, Huazhong Agricultural University, Wuhan, China

**Keywords:** potassium deficiency, endophyte community, nutrient uptake, *Sarocladium oryzae*, jasmonic acid

## Abstract

Sheath rot disease is an emerging fungal disease in rice, whose infection causes severe yield loss. *Sarocladium oryzae* (*S. oryzae*) is the major causal agent. Previous study has demonstrated that rice deficiency in potassium (K) aggravates *S. oryzae* infection. However, the effects of *S. oryzae* infection on the nutrient-uptake process, endophyte communities, and hormone level of host plant under K-deficiency condition remain unclear, the mechanism of K mediated *S. oryzae* infection needs to be further study. The present study analyzed alterations in the endophytic community and nutrient-uptake process of host plants through an exogenous inoculation of *S. oryzae* in pot and hydroponics experiments. *S. oryzae* infection sharply increased the relative abundance of *Ascomycota* and decreased the Shannon and Simpson index of the endophytic community. Compared with the K-sufficient rice infected with *S. oryzae*, K-starved rice infected with *S. oryzae* (−K + I) increased the relative abundance of *Ascomycota* in leaf sheaths by 52.3%. Likewise, the −K + I treatment significantly decreased the Shannon and Simpson indexes by 27.7 and 25.0%, respectively. Sufficient K supply increased the relative abundance of *Pseudomonas* spp. in the host plant. *S. oryzae* infection profoundly inhibited the nutrient uptake of the host plant. The accumulation of oleic acid and linoleic acid in diseased rice decreased the biosynthesis of jasmonic acid (JA), and the content of JA was lowest in the −K + I treatment, which suppressed K^+^ uptake. These results emphasize the importance of K in resistance to *S. oryzae* infection by modulating endophyte community diversity and enhancing the nutrient-uptake capacity of the host plant.

## Introduction

Rice (*Oryza sativa* L.) provides a staple diet for more than three billion people in the world, it is estimated that the yield of rice will need to double over the next 40 years to meet the food demands of the ever-expanding global population (Custodio et al., 2016). In China, rice planting area is approximately 3.0× 10^7^ hm^2^, accounting for about 30% of the total food crop planting area. However, the rice production was generally limited by heavy metal phytotoxicity (Yang et al., 2021), pre-harvest sprouting (Hu et al., 2016, 2017), nutrient deficiency, and disease infection (Liu et al., 2014), among others. Under the stress condition, microbial communities might mediate the tolerance capacity of host plant (Sheteiwy et al., 2021). According to the statistics, disease outbreaks account for approximately 30% of yield losses every year (Skamnioti and Gurr, 2009). Rice leaf sheath rot disease is a new type of fungal disease that has emerged in recent years and has occurred in major rice-producing regions around the world (Bigirimana et al., 2015). The typical symptom of this disease is that sheath rot lesions occur on the flag leaf sheath (FLS) of rice at the booting stage, which eventually results in leaf sheath rot as the lesion expands (Lanoiselet et al., 2012). The pathogens of rice leaf sheath rot disease are a kind of complex flora. Previous studies have shown that pathogens that can cause leaf sheath rot symptoms include *Sarocladium oryzae (S. oryzae)*, *Gibberella fujikuroi complex*, *Fusarium graminearum*, and *Pseudomonas fuscovaginae*, among others (Bigirimana et al., 2015). Among them, *S. oryzae* is the main pathogen. However, the effect of *S. oryzae* infection on the relative abundance of the endophytic community is still unclear. Therefore, screening the dominant causal agent of leaf sheath rot after *S. oryzae* infection and exploring effective strategies against *S. oryzae* infection are crucial for understanding the pathogenic mechanism and developing sustainable agriculture.

Endophytes are microbial communities that live in plants and have been detected in almost all higher plants, and they colonize plants mainly through wounds, roots, and stomata (Luo et al., 2012). There are large differences in the abundance of endophyte communities among different tissues and organs of the same plant (Leff et al., 2015). The changes in the endophyte community in rice stem and leaf tissue can reflect the ability of host plants to cope with stress conditions. Maintaining the balance of microorganisms in host plants is important for plant adaptation to adverse environments. Therefore, improving the stability and ecological diversity of plant endophytes can enhance the ability of host plants to tolerate stress conditions. Previous studies have constructed a database of endophytic bacterial 16S rDNA and fungal ITS rDNA sequences (Hardoim et al., 2015). By comparing the database, it was found that the plant endophyte community mainly includes bacterial sequences such as *Actinomycetes* and *Proteobacteria*, as well as fungal sequences such as *Pseudomonas* and *Ascomycota*. As a class of semisaprophytic pathogens, *S. oryzae* infects the leaf sheaths of rice, resulting in leaf sheath rot (Bigirimana et al., 2015), while the effects of *S. oryzae* infection on the endophytic community of the entire leaf sheaths are still unknown.

Potassium (K) is an essential mineral nutrient for rice growth. Our previous studies have demonstrated that a sufficient K supply decreased the yield loss of rice caused by *S. oryzae* infection, and the application of K increased the tolerance capacity of the host plant to *S. oryzae* (Zhang et al., 2019,^41,43^). K application increased the tolerance capacity of host plants to biotic stresses mainly by regulating the physiological metabolic process of host plants (Anschütz et al., 2014). However, physiological and metabolic changes in host plants are closely related to the functions of endophyte communities (Ma et al., 2021). Endophytes also regulate the composition and quantity of metabolic components in the host plant; for example, arbuscular mycorrhizal fungi can assist the host in stabilizing metabolism *in vivo* under adverse conditions (Yang et al., 2020). On the other hand, K nutrition and endophytes both regulated the hormone levels of host plants. Plants exposed to K deficiency induce the biosynthesis of oxylipins, which increases the expression of allene oxide synthase (*aos*) genes and jasmonic acid (JA) content (Troufflard et al., 2010). In turn, JA plays an important role in regulating the nutrient-absorption process, especially for K^+^ (Armengaud et al., 2004). Similarly, endophytes also mediate the stress tolerance capacity of host plants by regulating plant hormone levels. For example, some endophytes reduce the concentration of ethylene accumulated in plants by synthesizing 1-aminocyclopropane-1-carboxylate deaminase (Glick, 2014). However, the effects of K supply on endophyte communities and hormone levels of host plants during *S. oryzae* infection remain unknown. Thus, exploring the effects of K supply on endophyte communities and hormone levels of host plants during *S. oryzae* infection can enhance our understanding of the pathogenic mechanism of *S. oryzae*. In this study, the exogenous inoculation of *S. oryzae* was performed in pot and hydroponics experiments. The main objectives were to (1) explore the effects of *S. oryzae* infection on endophyte communities and nutrient uptake of host plants and (2) clarify the effect of K deficiency-induced variations in hormone levels on host nutrient uptake during *S. oryzae* infection. These results will help to further understand the pathogenic mechanism of *S. oryzae* infection.

## Materials and Methods

### Plant Materials and Experimental Design

#### Pot Experiment

A solution of 0.5% NaClO was used to disinfect the rice seeds (cv. Dongjing, Japan) for 8 h, after which the seeds were geminated in deionized water at 33°C for 2 days. Then, the germinated seeds were sown on a seedling tray (50%-strength nutrient solution without K^+^ supply) for 12 days of growth. Fourteen-day-old seedlings that were evenly growing were selected and used for a pot experiment. The soil material used in the pot experiment was collected from a long-term field experiment. The basic physical and chemical properties of the soil are as follows: pH 5.58 (1: 2.5, soil: deionized water), organic matter 34.3 g kg^−1^, total nitrogen 1.66 g kg^−1^, Olsen-phosphorus (P) 19.3 mg kg^−1^, slowly available K 320.2 mg kg^−1^, and NH_4_OAc-K 51.4 mg kg^−1^, which belongs to K-deficient soil. Two-factor randomized block design was adopted in the pot experiment with four treatments and six replicates, in total of 24 pots, each pot with total of 10 kg soil. Four treatments were as follows: (1) K-deficient treatment (−K), wherein no K fertilizer was applied in the pot and 10 μl of sterile water was inoculated on the FLS during the rice booting stage; (2) sufficient K supply treatment (+K), wherein the application rate of K in the pot was 1.5 g K_2_O/pot and 10 μl of sterile water was inoculated on the FLS during the rice booting stage; (3) no K fertilizer supply (−K+ I), wherein the FLSs were inoculated with 10 μl suspended spores of *S. oryzae* at a concentration of 1 × 10^7^ cells/mL during the rice booting stage (a hemocytometer was used to determine the spore concentration); and (4) K-sufficient plants with *S. oryzae* inoculation treatment (+K + I), wherein the application rate of K was 1.5 g K_2_O/pot and the *S. oryzae* was inoculated in FLSs during the booting stage. All the K fertilizers were in the form of potassium sulfate. To ensure that other nutrients would not limit rice growth, the supplies of nitrogen and phosphorus were 2.0 g N/pot soil and 1.0 g P_2_O_5_/pot, respectively. The forms of nitrogen and phosphorus were urea (N 46%) and superphosphate (P_2_O_5_ 12%). A total of 75% percent of urea and all the phosphate fertilizers were mixed with the soil as a base fertilizer application, and the remaining 25% percent of urea was topdressed with water at the jointing stage. Each pot was transplanted with two single seedlings 1 day after base fertilization.

#### Hydroponics Experiment

Two lines of *aos* (*aos1* and *aos3*), both serving as T-DNA insertion mutants, in the background of *Oryza sativa japonica* (cv. Dongjing) were used in the hydroponic experiment, and the seed materials were kindly provided by Professor Guozhang Kang from Henan Agriculture University. Homozygous plants of these two lines were identified using PCR as described previously (Li et al., 2017). The seeds of Dongjing, *aos1* and *aos3*, were sterilized with 0.5% NaClO for 8 h, rinsed with deionized water, and placed in a constant temperature incubator (33°C) for germination. After germination, the seeds were sown on hydroponic trays covered with gauze, and then, the gauze was immersed in deionized water and cultured in a glass greenhouse at 30°C for 8 days. Uniform seedlings were selected and placed in 6 l hydroponic buckets, and each bucket contained 5.5 l 25% strength nutrient solution. After 3 days, 50%-strength nutrient solution was supplied for another 3 days, and the 14-day-old seedlings were treated with strength nutrient solution (+K, 1 mm K_2_SO_4_) and strength nutrient solution with low potassium supply (−K, 0.01 mm K_2_SO_4_). The full-strength nutrient solution was composed as follows: 1.425 mm NH_4_NO_3_, 0.998 mm CaCl_2_, 0.323 mm NaH_2_PO_4_·2H_2_O, 1.643 mm MgSO_4_·7H_2_O, 1 mm K_2_SO_4_, 9.5 μm MnCl_2_·4H_2_O, 0.075 μm (NH_4_)_6_Mo_7_O_24_·4H_2_O, 0.019 mm H_3_BO_3_, 0.152 μm ZnSO_4_·7H_2_O, 0.155 nm CuSO_4_·5H_2_O, 0.125 mm FeSO_4_·7H_2_O 0.125 mm Na_2_EDTA·2H_2_O, and 0.250 mm Na_2_SiO_3_ 9H_2_O. The nutrient solution was changed every 3 days, and each treatment was replicated 5 times. The whole culture process was carried out in a light-transmitting glass greenhouse. During the culture, the humidity was 45–60%, the light intensity was 600 μmol m^−2^ s^−1^, the night temperature was 15°C, and the daytime temperature was 28°C.

### *S. oryzae* Inoculation

#### Pot Experiment

Rice flag leaf sheaths (FLSs) at the booting stage were selected for *S. oryzae* inoculation. The inoculation method was performed as described in our previous study (Zhang et al., 2021b). Briefly, 1× 10^7^ conidia mL^−1^ pathogen material was prepared by leaching a conidial suspension. Then, uniform FLSs with low K levels and appropriate K levels were selected using a punch to make a small hole in a FLS followed by injecting 10 μl of suspended spores. Sterile water at the same dose (10 μl) was injected as a control (mock inoculation). After inoculation, the pots were placed in a greenhouse with 90% humidity until successful infection (total of 12 h).

#### Hydroponics Experiment

4 weeks after commencing treatment, rice seeds of Dongjing, *aos1* and *aos3*, at the seedling stage were selected for *S. oryzae* inoculation. The inoculation method was the same as in pot experiment.

### Endophytic Microbial Community DNA Extraction

Typical brown lesions occurred on the FLSs after 5 days of inoculation in the pot experiment, and the mock inoculation treatment had no symptoms of infection. Representative FLSs for DNA extraction of endophytes were then collected. The surface sterilization of FLSs and the extraction of endophytes were carried out as suggested in a previous study (Araujo et al., 2002; Ruiz-Pérez and Zambrano, 2017). Briefly, (1) each FLS was washed with 70% alcohol for 1 min; (2) the FLS was washed with 2% NaClO for 3 min; (3) the FLS was washed with 70% alcohol for another 30 s; (4) the FLS was washed with sterile water for 30 s, 3–4 times in total; (5) to ensure the success of the sterilization process, 100 μl of the water used in the fourth step was spread on different agar plates; (6) the agar medium was incubated for 2 weeks in the dark in an incubator at 25°C; and (7) the water (described in step 4) was used for the first PCR labeling of the 16S rRNA gene or ITS region to control DNA-removal success. Surface-sterilized plant material was ground in liquid nitrogen using a sterile mortar and pestle, and the powdered plant material was stored at −20°C for DNA extraction. DNA was extracted using a soil DNA kit, which was extracted according to the manufacturer’s instructions, and the concentration and purity of DNA were detected by a NanoDrop One.

### Endophyte Community Analysis and Physiological Indicator Measurements

Using genomic DNA as a template, according to the selection of sequencing regions, PCR amplification was performed using primers with barcodes and Premix Taq (TaKaRa). Sixteen SV4 primers (515F and 806R) were used to identify bacterial and archaeal diversity; ITS1 primers (ITS5-1737F and ITS2-2043R) were used to identify fungal diversity. The PCR system is shown in Table 1.

**Table 1:** PCR reaction system.

Reagent name	Dosage (μl)
2x Premix Taq	25
Primer-F (10 mm)	1
Primer-R (10 mm)	1
DNA (20 ng/μl)	3
Nuclease-free water	20

GeneTools Analysis software (Version 4.03.05.0, SynGene) was used to compare the concentrations of PCR products, calculate the required volume of each sample according to the principle of equal mass, and mix the PCR products. An EZNA^®^ Gel Extraction Kit was used to recover PCR mixed products, and TE buffer elution was used to recover target DNA fragments. Library construction was performed according to the standard procedure of the NEBNext^®^ Ultra^™^ DNA Library Prep Kit for Illumina^®^. The constructed amplicon library was subjected to PE250 sequencing using the Illumina Hiseq2500 platform. Data analysis was performed according to the method suggested by Leff et al. (2015).

Typical symptoms of sheath rot appeared on FLSs 8 days after *S. oryzae* inoculation in the hydroponic experiment. At this time, fresh samples were collected from the shoots and roots of the treatment inoculated with *S. oryzae* and the mock treatment. The fresh samples used for comparing the sensitivity of WT and *aos* mutants to potassium deficiency were collected at 45 days after commencing K-starvation treatment. The collected samples were snap-frozen in liquid nitrogen and stored in a − 80°C ultralow temperature freezer. The internal standard 10-dihydro-JA (DHJA; OlChemIm) for JA measurement was purchased from Sigma, and the content of JA was determined according to the method described by Huang et al. (2021). The ultrahigh liquid chromatography-electrospray ionization tandem mass spectrometry method was used for the measurement of total JA content. The dry samples of shoots and roots were collected and dried in an oven at 65°C and then ground and passed through a 0.1 mm sieve. These dry samples were used for the determination of nutrients. Additionally, the relative contents of oleic acid and linoleic acid were measured through metabolome profiling as described in our previous study (Zhang et al., 2021b).

### Ionome Profiling

Shoot and root samples used for measurements of elemental contents were collected in the hydroponic experiment 8 days after *S. oryzae* infection. The inductively coupled plasma-mass spectrometry (ICP-MS) method suggested by McLoughlin et al. (2018) was used for the measurements of elemental contents. Briefly, 0.15 g of dry shoot and root samples were digested in a mixture of HNO_3_ and HClO_4_ (4 HNO_3_:1 HClO_4_), after which the digested solution was dissolved in 100 ml of ultra-pure water. Double filter paper was used to filter the solution, which was then used for ICP-MS profiling. Calibration curves were built based on a multielement standard (Ultra Scientific, Providence, RI, United States).

### Statistical Analyses

The measured parameters were analyzed by descriptive statistical analyses. Two-way analysis of variance was performed to reveal statistically significant differences among different treatments. Data analyses were performed in SPSS 19.0 (SPSS, Inc., Chicago, IL, United States). Graphics were created using Origin 9.0 software (OriginLab Corporation, Northampton, MA, United States). Community composition differences were assessed using permutation multivariate analysis of variance (PERMANOVA) with the vegan package in R 3.5.1.

## Results

### Species Community Differences at the Phylum Level After *S. oryzae* Infection Between K-Deficient and K-Sufficient Rice

**Figure 1:**
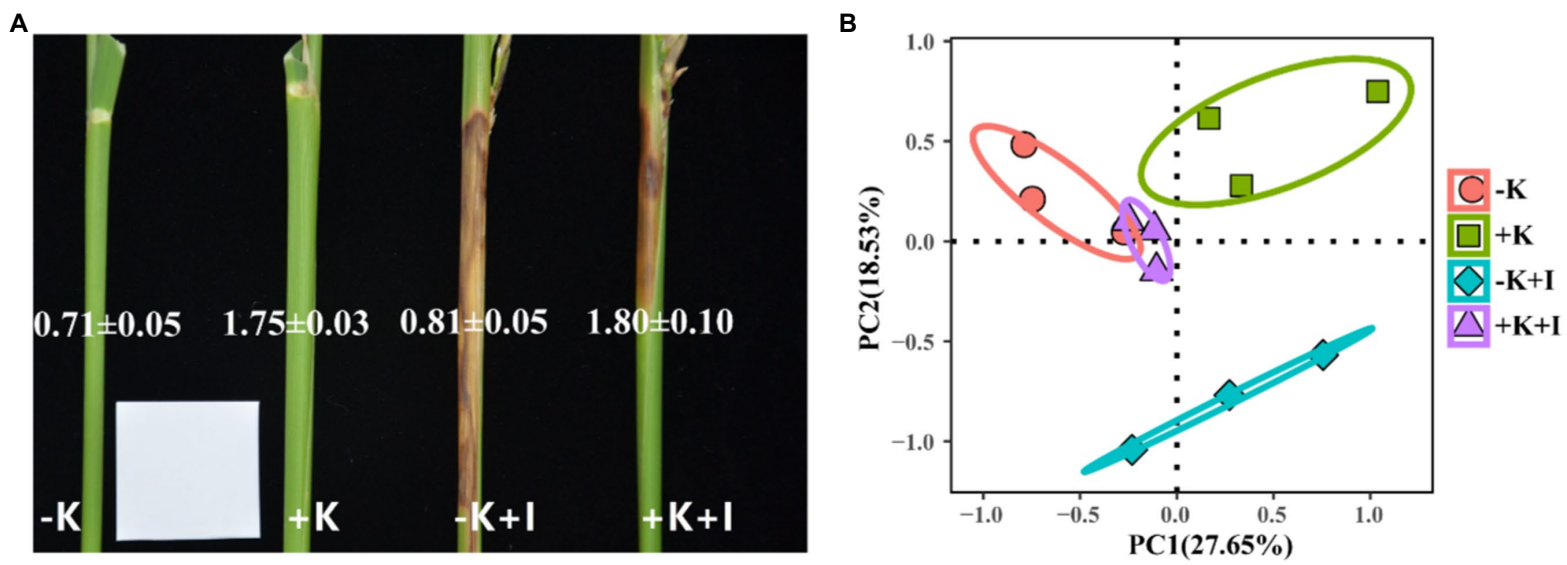
The lesions in flag leaf sheath **(A)** and principal component analysis of endophyte community in flag leaf sheath after *Sarocladium oryzae* inoculation **(B)**. Numbers in the picture represent K concentrations.

**Figure 2:**
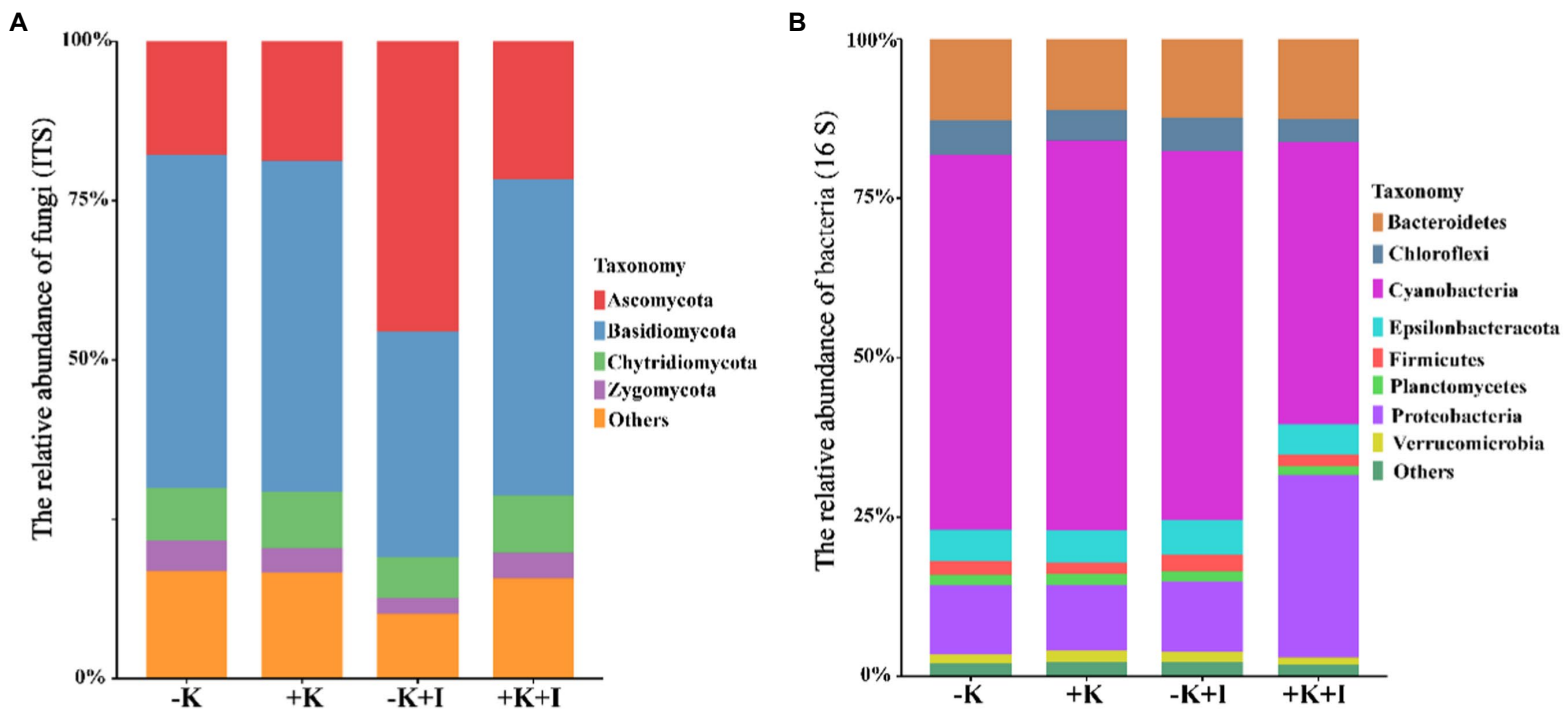
The relative abundance of fungi **(A)** and bacteria **(B)** in leaf sheath after *Sarocladium oryzae* inoculation. Different colors indicate different bacterial or fungal communities.

Potassium (K) deficiency promoted the infection of *S. oryzae*, which reflected that the largest length of the lesions occurred in the −K + I treatment (Figure 1A). Additionally, *S. oryzae* infection significantly altered the endophyte community structure in leaf sheaths. Principal component analysis (PCA) of the endophyte community showed that infection with *S. oryzae* significantly increased the differences in the endophyte community between K-sufficient and K-deficient rice (Figure 1B). Compared with the heathy plants, *S. oryzae* infection increased the relative abundance of *Ascomycota* by an average of 12.5%. Under the condition of *S. oryzae* inoculation, compared with the +K + I treatment, K deficiency increased the relative abundance of *Ascomycota* in leaf sheaths by 52.3% and reduced the relative abundance of *Basidiomycota* by 26.5% (Figure 2A). Similarly, *S. oryzae* infection also altered the bacterial communities in the flag leaf sheath (FLS). Compared with the −K + I treatment, the K supply significantly increased the relative abundance of *Proteobacteria* by 63.4%, and the relative abundance of *Cyanobacteria* was obviously decreased (Figure 2B). K deficiency profoundly affected the *alpha* diversity of the fungal community in the FLS during *S. oryzae* infection. Compared with the heathy plant, *S. oryzae* infection decreased the Shannon and Simpson indexes of fungi community by an average of 16.1 and 13.9%, respectively. Compared with the +K + I treatment, K-deficient rice infected with *S. oryzae* significantly decreased the Shannon and Simpson indexes of fungi community by 27.7 and 25.0%, respectively (Table 2).

**Table 2:** *Alpha* diversity of fungi community in flag leaf sheath after *Sarocladium oryzae* inoculation.

Treatment	Chao1	Dominance	Shannon	Simpson
−K	148.35 a	0.097 b	4.85 a	0.90 a
+K	132.83 a	0.105 b	4.72 a	0.89 a
−K + I	107.68 b	0.345 a	3.37 b	0.66 b
+K + I	133.63 a	0.101 b	4.66 a	0.88 a

*Different letters in the same column denote significant differences at *p* < 0.05 level, the same as below*.

### Species Cluster Analysis at the Genus Level After *S. oryzae* Infection Between K-Deficient and K-Sufficient Rice

**Figure 3:**
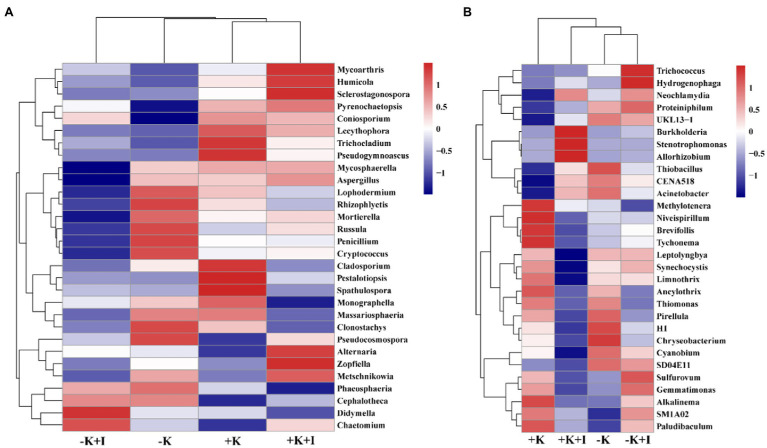
The clustering of fungal **(A)** and bacteria **(B)** species abundance after *Sarocladium oryzae* inoculation.

*S. oryzae* infection increased the differences in fungal and bacterial species at the genus level between K-deficient and K-sufficient rice. When compared with the +K + I treatment, K deficiency significantly increased the relative abundance of *Coniosporium, Chaetomium*, *Didymella*, and *Cephalotheca*, and all the other fungal species were decreased (Figure 3A). Compared with +K treatment, K-sufficient rice infected with *S. oryzae* significantly increased the abundance of *Burkholderia*, *Stenotrophomonas*, and *Allorhizobium* (Figure 3B). However, K deficiency profoundly decreased the *alpha* diversity of the bacterial community in the FLS during *S. oryzae* infection. The Chao1 index was used to reflect the diversity of the endophyte microbial community after rice leaf sheaths were inoculated with *S. oryzae*. Compared with the +K + I treatment, K-deficient rice infected with *S. oryzae* significantly decreased the Chao 1 index by 16.7% (Table 2).

### Effects of *S. oryzae* Infection on Linoleic Acid and Jasmonic Acid Content

**Figure 4:**
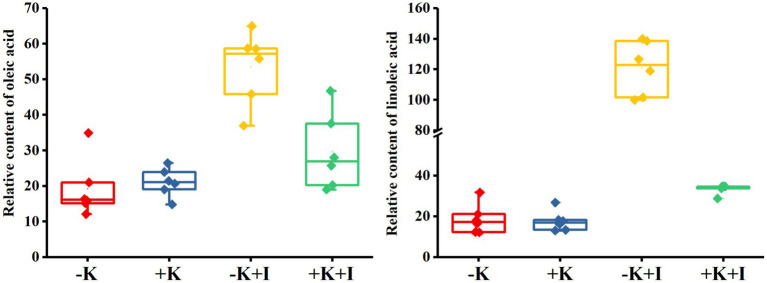
Relative content of oleic acid and linoleic acid content after *Sarocladium oryzae* inoculation.

**Figure 5:**
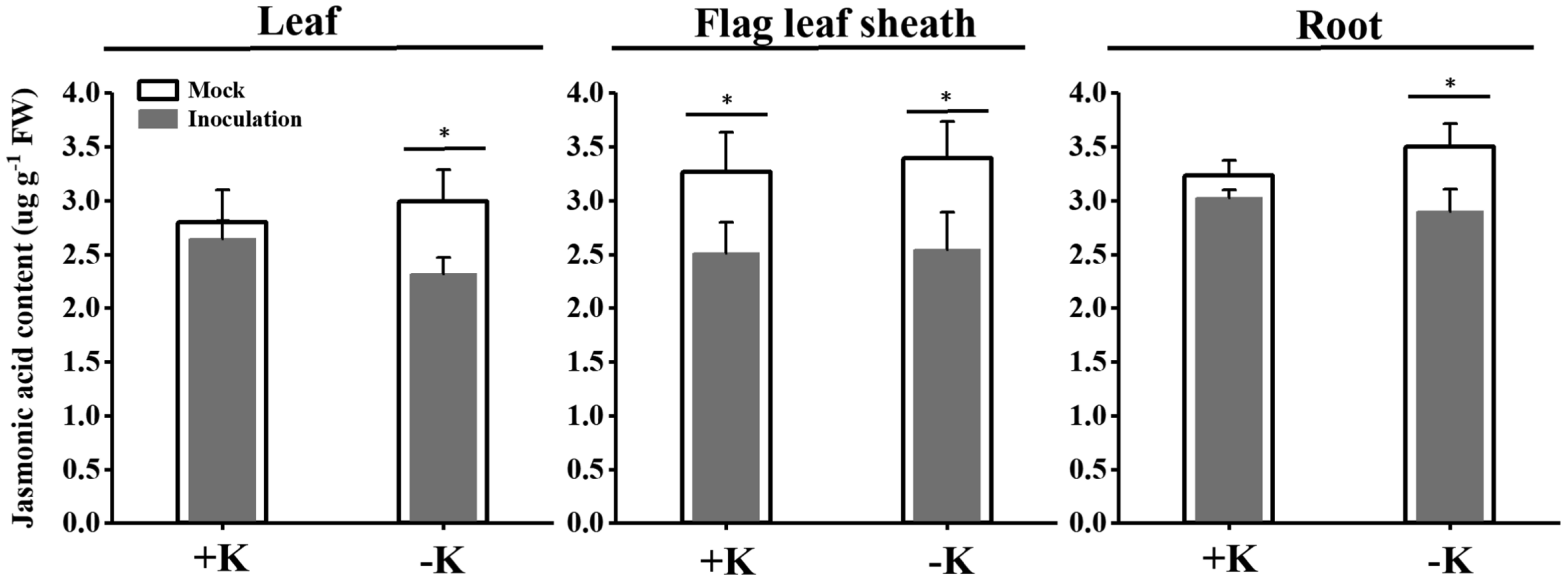
Effects of *S. oryzae* infection on jasmonic acid content among different rice tissues. ^*^Denotes a significantly differences between mock and inoculation treatments (*p* < 0.05).

*S. oryzae* infection significantly affected oleic acid and linoleic acid metabolic processes in FLS. Compared with the heathy plant, *S. oryzae* infection increased the relative content of oleic acid and linoleic acid by an average of 127.4 and 326.3%, respectively. Compared with +K + I treatment, K-deficient rice infected with *S. oryzae* increased the oleic acid and linoleic acid content by 82.0 and 261.5%, respectively (Figure 4). Oleic acid and linoleic acid are the synthetic precursors of jasmonic acid (JA), which is initially synthesized from linoleic acid and is localized in chloroplasts. *S. oryzae* infection decreased the JA content of leaves, FLSs, and roots by averages of 14.3, 24.1, and 12.7%, respectively. Notably, K deficiency amplifies the effect of *S. oryzae* infection on JA content. Compared with −K-treated rice, K-deficient rice infected with *S. oryzae* decreased the JA contents of leaves, FLSs, and roots by 22.5, 24.9, and 17.1%, respectively, while the corresponding values of +K + I treatment were decreased by 5.5, 23.1, and 6.6%, respectively (Figure 5).

### Effects of *aos* Gene Mutation and *S. oryzae* Infection on Nutrient Uptake in Rice

**Figure 6:**
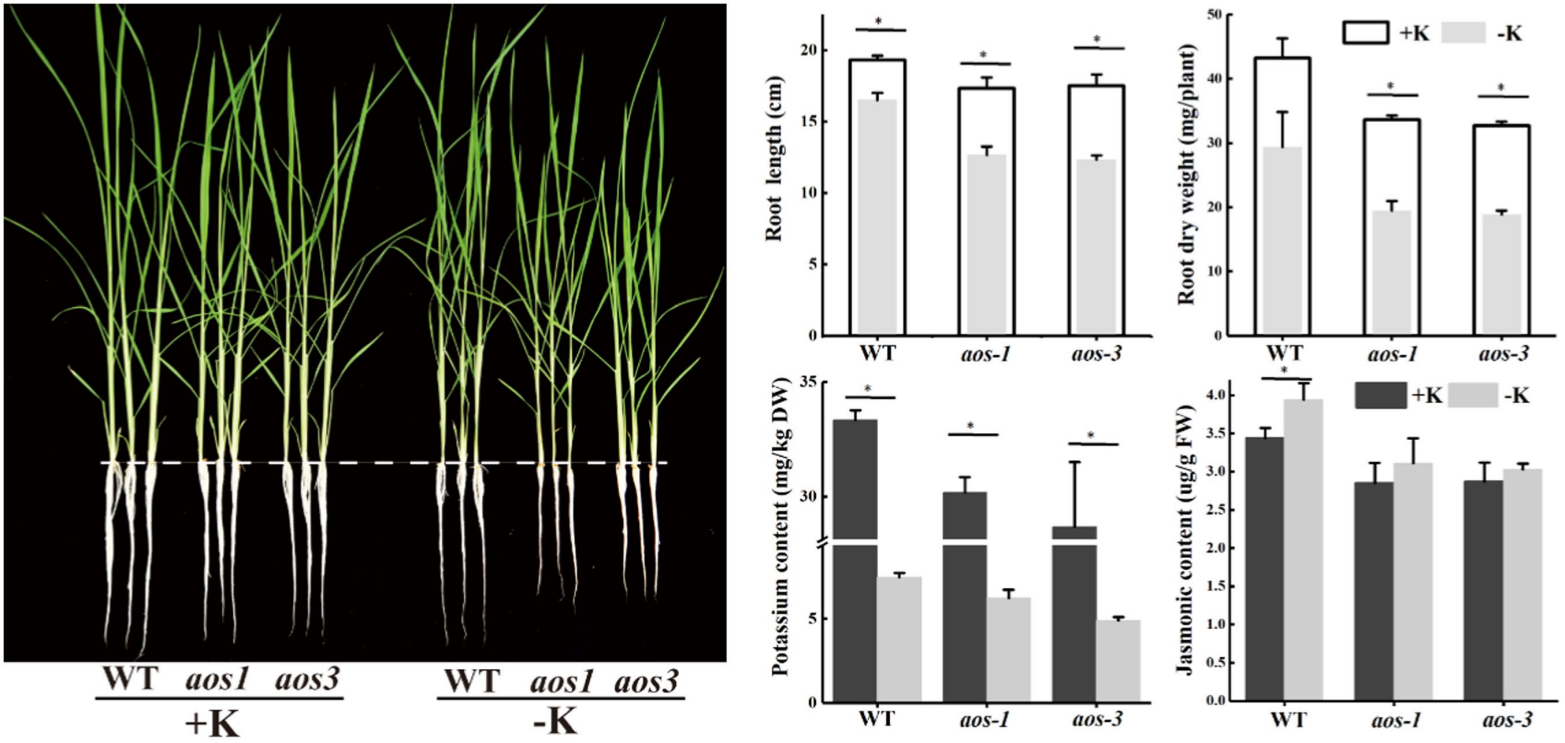
*aos* mutants show more sensitive to potassium deficiency. ^*^Denotes a significantly differences between −K and + K treatments (*p* < 0.05).

**Figure 7:**
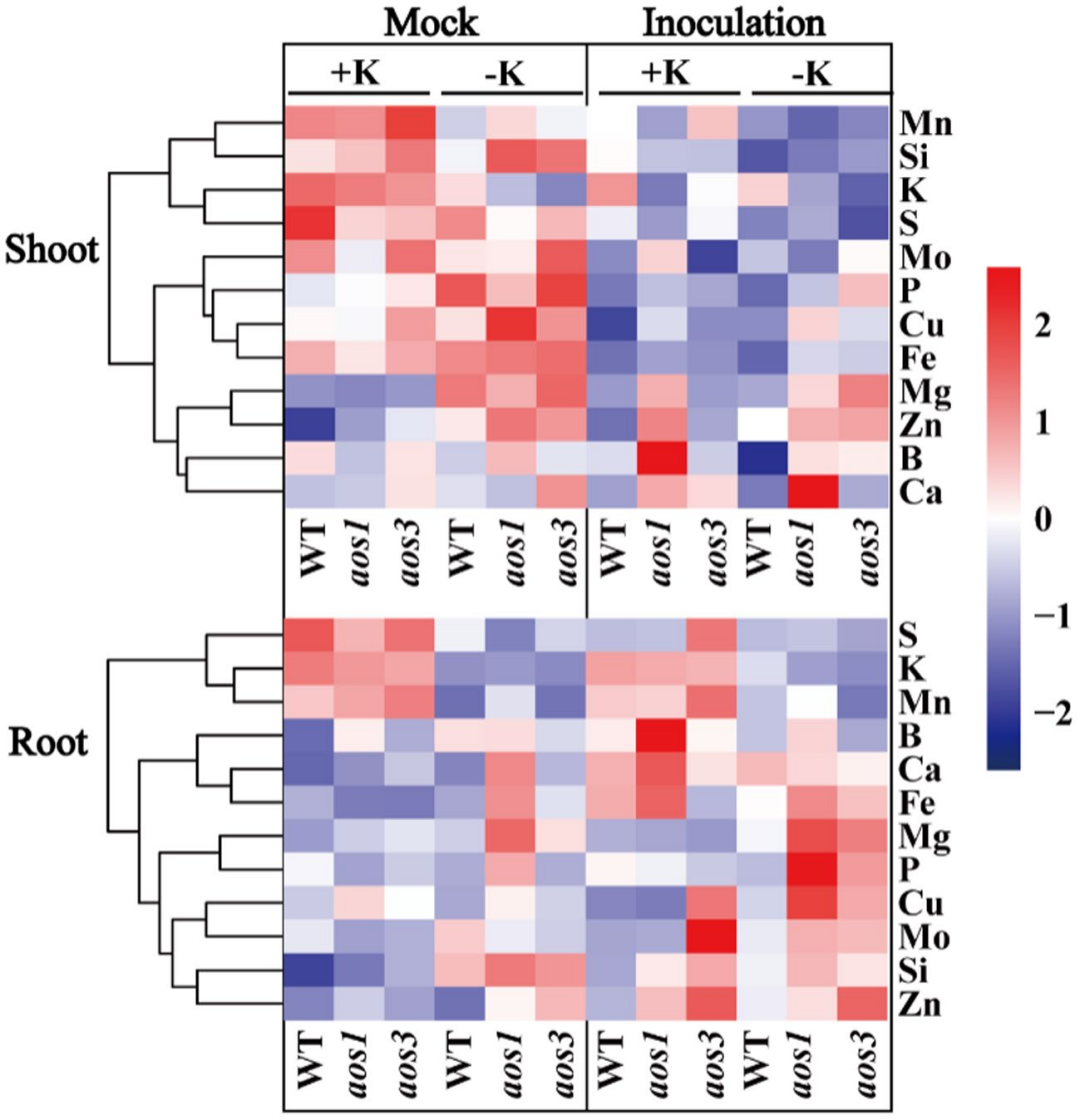
Effects of *S. oryzae* infection on nutrients uptake in rice.

Allene oxide synthase (*aos*) is the key enzyme that controls the synthesis of JA, and *aos* mutants show significantly lower JA content than the wild type (WT), especially under the condition of K deficiency. Additionally, *aos* mutants are more sensitive to potassium deficiency. The root length and the root dry weight of *aos1* and *aos3* were obviously lower than those of WT under the condition of K deficiency (Figure 6). *aos* gene mutation also inhibited K^+^ uptake. Compared with WT, the K^+^ content of *aos1* and *aos3* decreased by averages of 10.7 and 17.7%, respectively. ICP-MS was used to analyze the changes in the ionomes of roots and shoots after *S. oryzae* infection (Figure 7). The results demonstrated that *S. oryzae* infection resulted in a significant decrease in nutrient uptake. *S. oryzae* infection significantly reduced the contents of K, Si, S, Mo, and Fe in the shoots. In contrast, the infection increased the content of most nutrients in the root parts, such as Ca, Fe, Mg, and Cu. Compared with the K-sufficient treatment (+K), K deficiency resulted in lower S, K, and Mn contents in roots under both diseased and healthy conditions. Likewise, K deficiency decreased the contents of Mn, K, Si, and S in the shoots of diseased plants, while the contents of these nutrients exhibited no significant differences in healthy plants.

## Discussion

**Figure 8:**
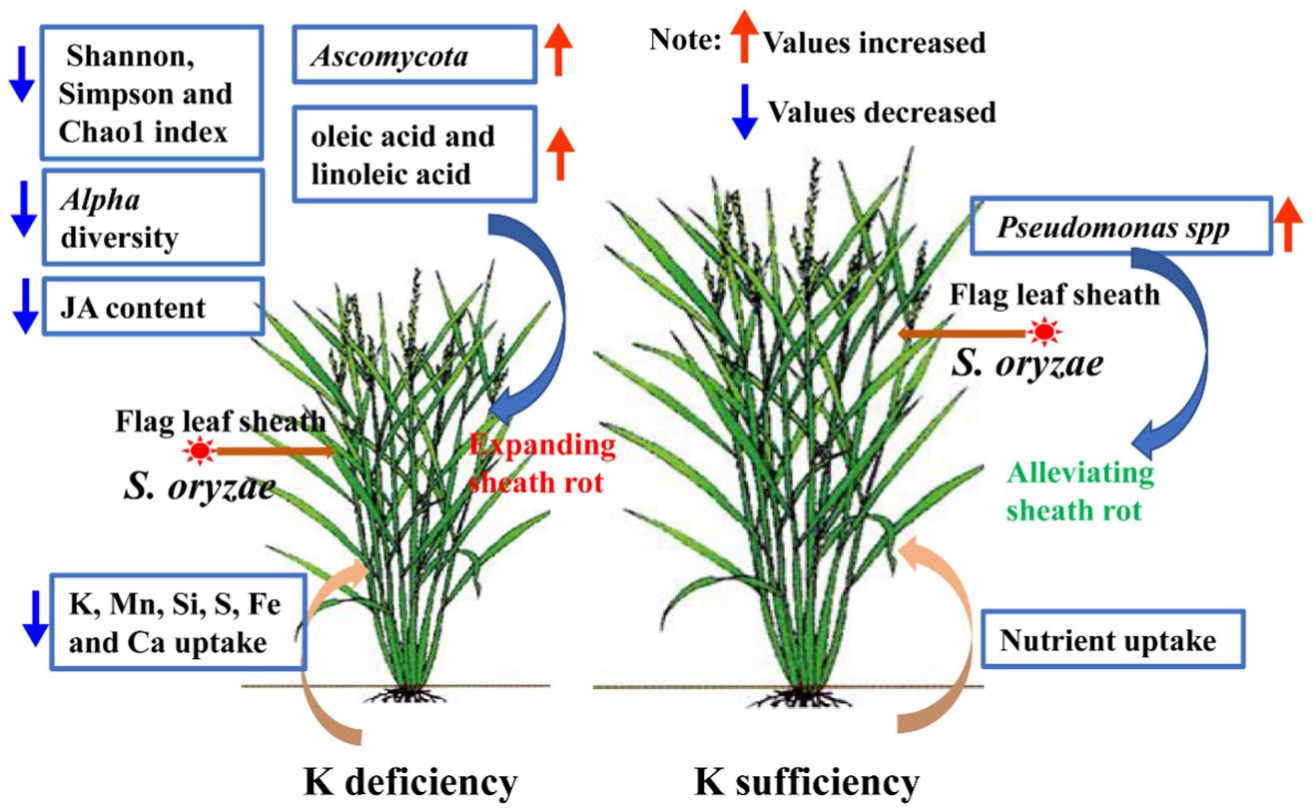
Summary model. *S. oryzae* infection alters the metabolism process, which reflected as the accumulation of oleic acid and linoleic acid in flag leaf sheath. K starvation promoted *S. oryzae* infection and linoleic acid accumulation. In turn, the alterations in metabolites of host plant affect the growth of endophyte communities. Meanwhile, due to the linoleic acid is the precursor for JA synthesis, hyper-accumulation of linoleic acid in leaf sheath causes the decreasing in JA content. JA mediated the nutrients uptake process, especially for K^+^ uptake; thus, the low level of JA further inhibited the nutrients uptake of host plant. Finally, K-starvation aggravates *S. oryzae* infection and ultimately leads to alterations in endophyte communities and suppression of nutrient uptake.

In this study, K deficiency in rice aggravates *S. oryzae* infection and ultimately leads to alterations in endophyte communities and suppression of nutrient uptake, especially for K^+^ uptake, the specific mechanism and nutrient-uptake process are shown in Figure 8. Below we will discuss the alterations in endophyte communities and nutrients uptake of K-starved rice in response to *S. oryzae* infection.

### The Causal Agent of Sheath Rot and Its Pathogenic Mechanism Underlying

The causal agent of sheath rot disease was first identified as *Acrocylindrium oryzae* in Taiwan in 1922 (Mew and Gonzales, 2002). *Acrocylindrium oryzae* infection induces the typical symptoms of leaf sheath rot and causes server yield loss. In 1975, researchers formally established the genus *Sarocladium*, which covers potential pathogens, such as phytopathogens, mycoplasmas, and saprophytes (Gams and Hawksworth, 1975; Giraldo et al., 2015). According to the classification, *S. oryzae* belongs to *Hypomycetes* and *Ascomycota*. Previous studies have demonstrated that phytotoxins such as cerulenin produced by *S. oryzae* can inhibit the development of other fungi (Gnanamanickam and Mew, 1991). Consistent with our result, *S. oryzae* infection significantly decreased the abundance of other endophytic fungi in FLSs (Figure 2; Tables 2 and 3). Additionally, helvolic acid secreted during *S. oryzae* infection shows strong antibacterial activity against Gram-positive bacteria (Tschen et al., 1997). A similar phenomenon was observed in the present study: the abundance of endophytic bacterial communities in leaf sheaths obviously decreased after *S. oryzae* infection, especially in K-deficient rice (Figure 3). During the infection process, *S. oryzae* will interact with other pathogenic microorganisms, such as the synergistic effect of pathogenic bacteria and other mites, which results in sterile grains (Karmakar, 2008; Hummel et al., 2009). Moreover, bacterial sheath brown rot caused by *Gluconococcus rhizogenes* may also complicate *S. oryzae* infection, which ultimately results in leaf sheath rot (Venkataraman et al., 1987). All these results demonstrate that the infection process of pathogens that cause leaf sheath rot is complex. Our results further confirm that *S. oryzae* is the major pathogenic fungi of sheath rot disease, and it also interacts with other endophytes. After *S. oryzae* successfully invaded the leaf sheath, the pathogens inhibited the growth of other endophytic fungi and their ability to obtain ecological niches, which was conducive to its rapid colonization and finally causes sever yield loss.

**Table 3:** *Alpha* diversity of bacterial community in flag leaf sheath after *Sarocladium oryzae* inoculation.

Treatment	PD whole tree	Chao1	Dominance	Shannon	Simpson
−K	14.00 a	140.5 b	0.071 b	4.90 a	0.93 a
+K	13.00 a	154.1 a	0.079 b	4.79 a	0.92 a
−K + I	13.60 a	128.0 c	0.078 b	4.84 a	0.92 a
+K + I	13.30 a	153.8 a	0.096 a	4.70 a	0.90 a

### Potassium Deficiency Enlarged the Negative Impacts of *S. oryzae* Infection on the Endophyte Community

*S. oryzae* infection alters the endophyte community of the leaf sheath predominantly by secreting phytotoxin and altering the metabolism process of host plant, while the K level of the leaf sheath mediated those process. A previous study showed that K deficiency increased the competition capacity of pathogens for nutrients (Holzmueller et al., 2007). In the present study, the relative abundance of *Ascomycota* was sharply increased during the infection; therefore, *S. oryzae* shows a strong competitive capacity for niches in the K-starved leaf sheaths. Notably, *Pseudomonas* spp. shows the potential to inhibit the infection of *S. oryzae* (Surya et al., 2019). In the present study, sufficient K supply significantly increased the abundance of *Pseudomonas* spp. during *S. oryzae* infection. Thus, it was speculated that *Pseudomonas* spp. might play an important role in K-sufficient rice against *S. oryzae* infection. However, the mechanism of antagonism between pathogenic bacteria and endophytes needs to be further studied. Because the growth rate of *S. oryzae* in K-deficient leaf sheaths was faster than that in K-sufficient rice, the development of *S. oryzae* preempted the niche for other fungi, which explains why the endophyte community abundance was lowest in the −K + I treatment (Tables 2 and 3). The decreased diversity of the community also indicated the instability of the community, this result supports our hypothesis mentioned in the introduction that K deficiency reduces endophyte community stability in diseased leaf sheaths, which was beneficial for the colonization and development of *S. oryzae*.

### *S. oryzae* Infection Suppresses Nutrient Uptake by the Host Plant by Regulating Jasmonic Acid Levels

During the interaction between host plants and microorganisms, the host plants provide a stable environment, nutrients, and energy for the survival of microorganisms, while the microorganism directly or indirectly impacts the host plant through its own metabolites (Dreyfuss and Chapela, 1994). The colonization of endophytes will inevitably cause numerous responses of the host plant, including stimulation of plant defense responses, gene expression, and metabolism, among others (Pang et al., 2018). Our previous study also demonstrated that *S. oryzae* infection profoundly alters the metabolic process of host plants, especially the lipid metabolism process, the infection of which significantly increases the accumulation of lipid metabolism products (Zhang et al., 2021b). Similarly, based on our data, *S. oryzae* infection sharply increased the accumulation of oleic acid and linoleic acid, and the linoleic acid metabolism pathway was predominantly affected by *S. oryzae* infection (Figure 4; Supplementary Figure S1), which is consistent with a previous study showing that *S. oryzae* infection inhibited fatty acid synthesis by secreting cerulenin (Bigirimana et al., 2015). Because linoleic acid is the precursor for JA synthesis, its accumulation could suppress the JA synthesis process. However, JA is crucial for nutrient uptake, especially for K^+^ and boron, among others (Armengaud et al., 2004; Huang et al., 2021). Thus, the low JA level caused by *S. oryzae* infection (especially for K-starved rice) significantly suppressed the uptake of K^+^ (Figure 7), which might be due to the expression of K^+^ uptake-related genes was inhibited by low JA level (Supplementary Figure S2). Additionally, ionomics-detection technology has been used in the study of plant diseases and the interaction of plants and fungi. Ramos et al. (2009) used ion-selective electrode technology to explore the interaction between fungi and *Arabidopsis thaliana* and found that Ca^2+^ has a signaling role in fungi promoting ion uptake in plant roots. Li et al. (2012) used ion scanning electrode technology to confirm that ectomycorrhizal fungi regulated the Na^+^/K^+^ balance in poplar, which improved the salt tolerance of salt-sensitive poplars. K^+^ is the most abundant cation in higher plants and plays an important role in maintaining the electrochemical balance of the cytoplasm, catalyzing enzymatic reactions, regulating the osmotic balance of cells, and maintaining the turgor pressure of cells (de Bang et al., 2021). A previous study indicated that the fungal pathogen *Magnaporthe oryzae* suppresses the K^+^ absorption process (Shi et al., 2018). Similarly, in the present study, we found that *S. oryzae* infection not only significantly reduced the absorption capacity of K^+^ but also suppressed the uptake of other nutrients by the root system (Figure 7). This process might correlate with the inhibition of JA in the host plant. *S. oryzae* infection significantly decreased the JA content of leaves, FLSs, and roots in this study (Figure 5). Finally, low JA levels in the host plant significantly decreased the uptake of Mn, Si, S, Fe, and Ca (Figure 7). *aos* mutation significantly decreased the JA content of host plants, which makes rice more sensitive to K-deficiency stress (Li et al., 2017). In this study, we found that the *aos* mutation significantly suppressed the expression of K^+^ uptake channel genes such as OsAKT1 and OsHAK5 (Supplementary Figure S2). Under the condition of K deficiency, *aos* mutants exhibited the weakest K^+^ absorption capacity, which exacerbated the negative impacts of *S. oryzae* infection on K^+^ uptake by the host plant (Figure 7).

## Conclusion

*S. oryzae* infection sharply increased the relative abundance of *Ascomycota*, which decreased the Shannon and Simpson indexes of the endophytic community. Rice deficiency in K promoted the infection of *S. oryzae* and decreased the relative abundance of the endophytic community. Sufficient K supply increased the relative abundance of *Proteobacteria, Burkholderia*, *Stenotrophomonas*, *Allorhizobium, and Pseudomonas* spp. under *S. oryzae* infection conditions. *S. oryzae* infection profoundly suppressed the nutrient-uptake capacity of the host plant, including K, Mn, Si, S, Fe, and *Ca*. Additionally, the infection increased the accumulation of oleic acid and linoleic acid, which simultaneously decreased the biosynthesis of JA. K deficiency enlarged the decreasing range of JA under the condition of *S. oryzae* infection, which further inhibited K^+^ uptake.

## Data Availability Statement

The original contributions presented in the study are included in the article/Supplementary Material, further inquiries can be directed to the corresponding author.

## Author Contributions

The experiment was designed and performed by JZ, XL, ZL, RC, TR and JL. JZ wrote the original draft. All authors contributed to the article and approved the submitted version.

## Funding

This work was financially supported by the National Natural Science Foundation of China (31872174).

## Conflict of Interest

The authors declare that the research was conducted in the absence of any commercial or financial relationships that could be construed as a potential conflict of interest.

## Publisher’s Note

All claims expressed in this article are solely those of the authors and do not necessarily represent those of their affiliated organizations, or those of the publisher, the editors and the reviewers. Any product that may be evaluated in this article, or claim that may be made by its manufacturer, is not guaranteed or endorsed by the publisher.
