# Supplementary material for: Potassium Deficiency in Rice Aggravates Sarocladium oryzae Infection and Ultimately Leads to Alterations in Endophyte Communities and Suppression of Nutrient Uptake
Source: Front Plant Sci. 2022 Apr 26;13:882359. doi: 10.3389/fpls.2022.882359 (PMC9087805; doi:10.3389/fpls.2022.882359)
Supplement: Supplementary file 1 [file Presentation_1.pdf]

Potassium deficiency in rice aggravates Sarocladium oryzae infection and ultimately leads to alterations in endophyte communities and suppression of nutrient uptake

Jianglin Zhang [1], Zhifeng Lu [1], Rihuan Cong [1], Tao Ren [1], Jianwei Lu [1], Xiaokun Li [1,2*]

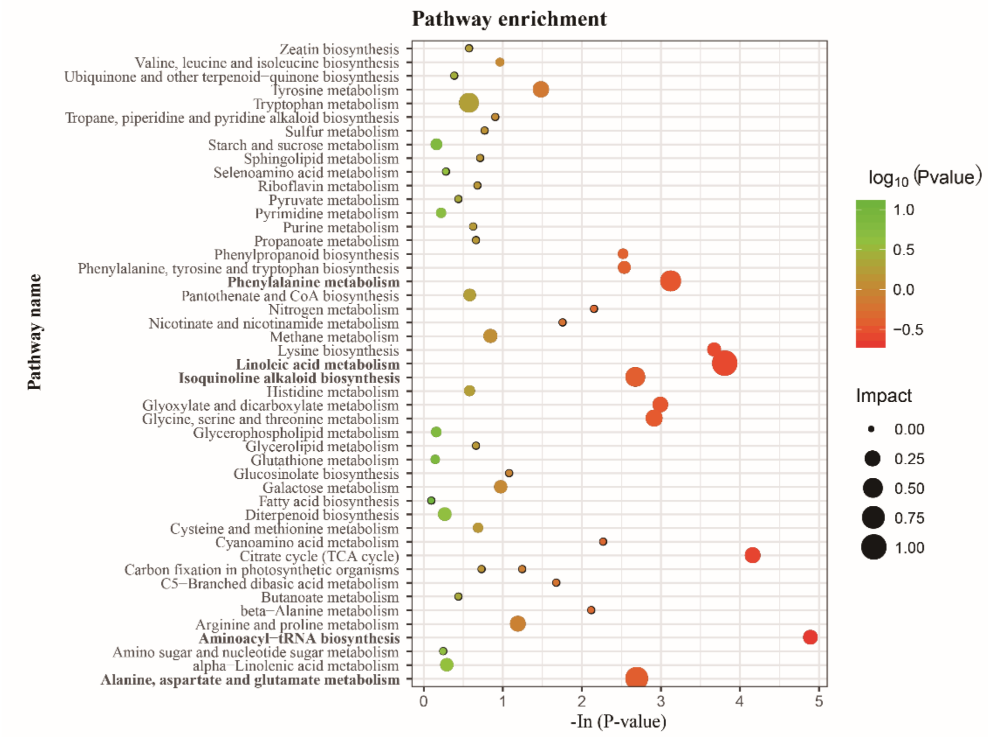

Fig S1 The major metabolism pathway of host plant after *S. oryzae* infection (K deficient rice versus K sufficient rice under the condition of *S. oryzae* infection).

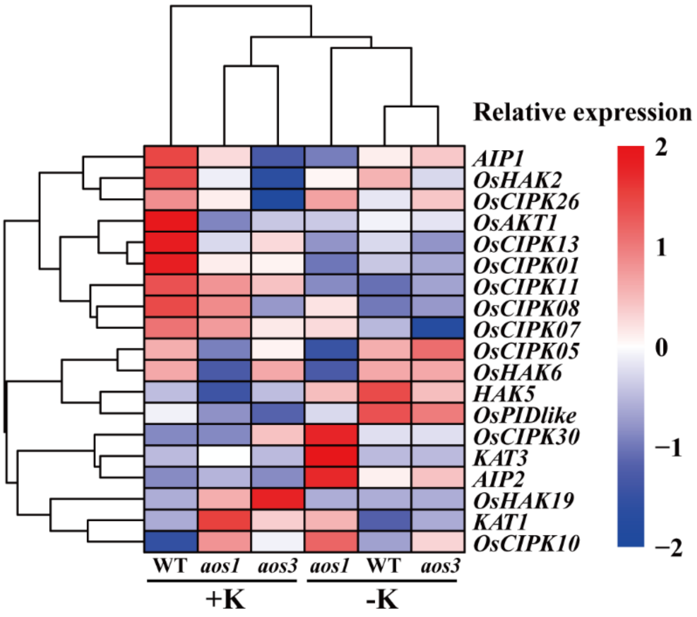

Fig S2 Effects of potassium deficiency and aos mutation on the expression of potassium uptake-related genes in rice
